# Chidamide: Exploration of maintenance therapy for patients with DLBCL with HBV infection

**DOI:** 10.1016/j.isci.2025.114302

**Published:** 2025-12-01

**Authors:** Ying Zhang, Haotian Wang, Wei Guo, Yangzhi Zhao, Zhumei Zhan, Zhe Li, Bowen Wang, Ou Bai

**Affiliations:** 1 Department of Hematology, The First Hospital of Jilin University, Changchun, China; 2 Department of Hematology, Shandong Provincial Hospital Affiliated to Shandong First Medical University, Jinan, Shandong 250021, China

**Keywords:** Microbiology, Viral microbiology

## Abstract

Hepatitis B virus (HBV) infection worsens the prognosis of patients with diffuse large B-cell lymphoma (DLBCL), possibly through epigenetic mechanisms. We conducted a prospective, single-arm, open-label clinical trial (NCT04661943) to evaluate the efficacy and safety of chidamide maintenance therapy in patients with HBV+ DLBCL who achieved complete or partial response after first-line treatment. Seventy patients with HBV+ were enrolled, including 30 receiving chidamide (20 mg twice weekly for two years) and 40 controls who declined treatment, with a propensity score-matched HBV- cohort for comparison. Chidamide maintenance significantly improved progression-free survival (*p* = 0.035) and overall survival (*p* = 0.041) in patients with HBV+ and was well tolerated without unexpected adverse events. These findings indicate that chidamide maintenance therapy can overcome HBV-associated poor prognosis in DLBCL and may serve as an effective maintenance strategy for this high-risk group.

## Highlights

• Chidamide improves OS and PFS in patients with HBV+ DLBCL post-CR/PR
• HBV infection is an independent adverse factor for OS in DLBCL
• Chidamide provides therapeutic benefit for patients with HBV+ DLBCL
• Chidamide was well tolerated with no unexpected adverse events

## Graphical abstract

**Figure:**
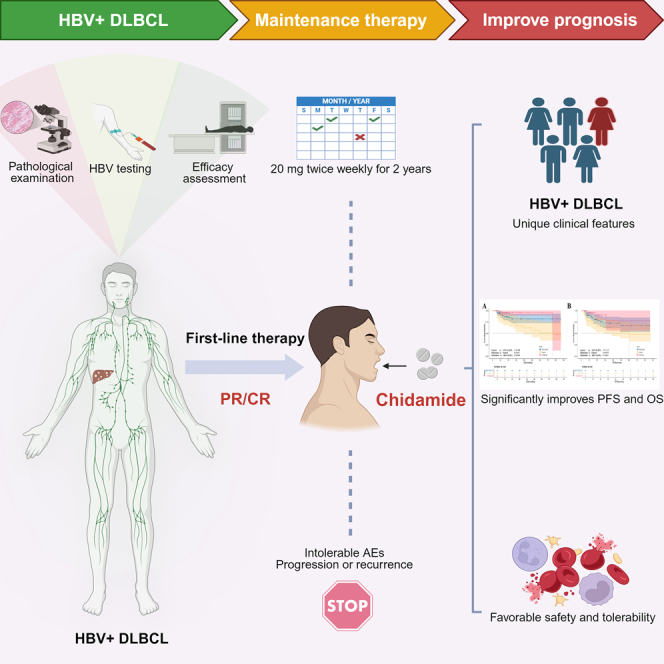

## Introduction

Viral infections are considered important inducers of many diseases, especially cancer.^1^^,^^2^ Over the past decade, epidemiological studies have consistently demonstrated a strong association between hepatitis B virus (HBV) infection and the development of non-Hodgkin lymphoma (NHL),^3^^,^^4^^,^^5^ with chronic HBV infection and naturally acquired HBV immunity increasing the risk of B-cell NHL.^6^ Diffuse large B-cell lymphoma (DLBCL) has been identified as the most common NHL subtype in patients with chronic HBV infection, confirmed in multiple retrospective studies and meta-analyses.^7^^,^^8^^,^^9^

The pathogenesis of virus-associated lymphomas has been partially elucidated. Proposed mechanisms include the direct infection and transformation of lymphocytes by oncogenic viruses, chronic B-cell activation induced by viral antigens or soluble factors, and immune evasion under prolonged immunodeficiency, which collectively contribute to the emergence of malignant clones.^10^ However, obtaining a comprehensive understanding of viral oncogenesis remains challenging, largely due to the intricate biological characteristics of the viruses themselves and the technical difficulties in establishing reliable animal models.^10^ Although the fifth edition of the World Health Organization Classification of Haematolymphoid Tumors does not classify DLBCL with concurrent HBV infection as a distinct subtype, accumulating evidence suggests that this presentation possesses unique clinical and prognostic characteristics. Evidence consistently shows that patients with HBsAg^+^ NHL, especially those with DLBCL, tend to be younger at diagnosis, present at a more advanced stage, exhibit elevated lactate dehydrogenase (LDH), have more frequent B symptoms, and demonstrate increased liver and spleen involvement.^11^^,^^12^^,^^13^^,^^14^ Patients with DLBCL with chronic HBV infection have significantly worse 2- and 5-year overall survival (OS) and progression-free survival (PFS) outcomes.^11^^,^^15^ The introduction of rituximab has markedly improved the prognosis of patients with DLBCL.^16^ However, in patients with HBsAg^+^, studies have demonstrated no significant difference in survival outcomes between those treated with R-CHOP and those receiving CHOP, suggesting that the addition of rituximab may not fully overcome the adverse effect of HBV infection.^12^^,^^17^ Moreover, HBV-related chemoresistance has been increasingly recognized, potentially contributing to inferior treatment responses and long-term outcomes in this population.^18^^,^^19^ These findings suggest that HBV infection may confer greater biological aggressiveness on DLBCL and reduce its responsiveness to conventional immunochemotherapy such as R-CHOP. However, current standard treatment strategies have shown limited efficacy in this patient population and remain insufficient to meet their clinical needs.

Maintenance therapy is a critical component of cancer treatment strategies; the primary goal is to consolidate remission achieved with the initial therapy, delay disease relapse, suppress the expansion of drug-resistant clones, and ultimately improve both disease-free survival (DFS) and OS outcomes.^20^ In the context of HBV-associated DLBCL, underlying immune dysregulation, persistent viral replication, and impaired B-cell function collectively contribute to higher relapse rates, increased treatment resistance, and inferior clinical outcomes. These disease-specific challenges highlight the urgent need for maintenance strategies specifically tailored to this high-risk patient population.

Epigenetic dysregulation is a well-established hallmark of hematologic malignancies, representing an attractive therapeutic target. Although HBV is known to participate in epigenetic modulation, existing research has predominantly focused on its role in hepatocellular carcinoma, and its effect on lymphomagenesis remains underexplored.^21^^,^^22^^,^^23^ Chidamide is the first orally active, selective histone deacetylase inhibitor (HDACi) approved in China. This drug was initially approved by the China Food and Drug Administration in 2014 for the treatment of relapsed or refractory peripheral T cell lymphoma (PTCL); in 2024, it was approved for the treatment of previously untreated double-expressor (MYC^+^/BCL2^+^) DLBCL.^24^ Numerous studies have demonstrated that chidamide limits tumor progression by modulating the tumor microenvironment, inducing cell-cycle arrest, promoting apoptosis, regulating autophagy, and enhancing complement-dependent and antibody-dependent cell-mediated cytotoxicity.^24^^,^^25^ Given HBV’s involvement in epigenetic alterations, the use of chidamide as a maintenance therapy offers a biologically rational and potentially impactful approach for patients with DLBCL with HBV coinfection.

Building upon this rationale, we conducted a prospective, single-center clinical study at our institution involving newly diagnosed patients with DLBCL with concurrent HBV infection. Patients who achieved complete response (CR) or partial response (PR) following first-line therapy were administered chidamide as maintenance treatment. Through systematic longitudinal follow-up assessing both efficacy and safety endpoints over a defined period, we comprehensively evaluated the therapeutic potential of chidamide in this high-risk population. Our findings provide robust clinical evidence that can inform the management of HBV-associated DLBCL and establish a foundation for future investigations into epigenetically targeted therapies tailored to virus-associated lymphomas.

## Results

**Figure 1:**
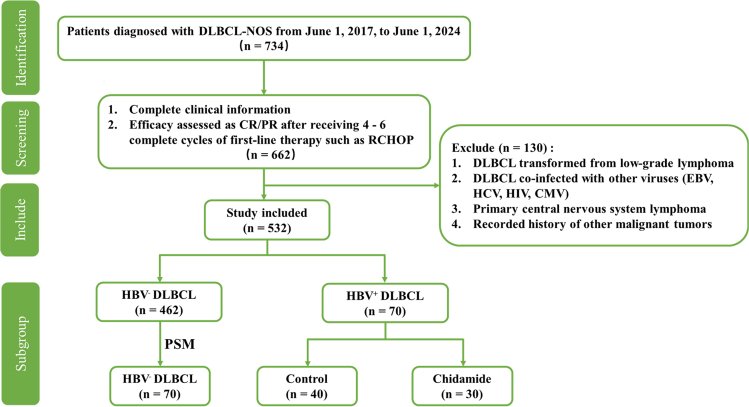
Flowchart of the study

Between June 1, 2017, and June 1, 2024, a total of 734 patients with newly diagnosed DLBCL were admitted to our center. After applying the predefined inclusion and exclusion criteria, 532 patients were eligible for inclusion, comprising 70 with HBV^+^ DLBCL and 462 with HBV^−^ DLBCL. Among the 70 patients with HBV^+^ DLBCL, 30 received chidamide maintenance therapy (chidamide group), while 40 declined (control group). To minimize potential confounding factors and enhance comparability between groups, a 1:1 PSM method was applied to select a matched cohort of patients with HBV^−^ DLBCL (HBV^−^ DLBCL group) from the 462 eligible patients with HBV^−^. The flowchart of patient enrollment, grouping, and the PSM process is shown in Figure 1.

### Baseline characteristics

The baseline characteristics of all patients are summarized in Table 1. Patients with HBV^+^ DLBCL exhibited significantly higher rates of abnormal liver function (*p =* 0.046), spleen involvement (*p <* 0.001), and bone marrow involvement (*p =* 0.002) versus patients with HBV^−^ DLBCL. Additionally, a greater proportion of patients with HBV^+^ DLBCL had more than two extranodal sites involved (*p =* 0.027). However, no significant differences were observed between the two groups in terms of age, sex, B symptoms, ECOG score, International Prognostic Index (IPI) score, LDH level, β2-MG level, cell origin, pathological features, bulky disease, or other clinical characteristics.

**Table 1:** Clinical characteristics of patients with HBV^+^ and HBV^−^ DLBCL before and after PSM

Variables	Before PSM			After PSM		
	HBV^+^ DLBCL (*n* = 70)	HBV^−^ DLBCL (*n* = 462)	*p*	HBV^+^ DLBCL (*n* = 70)	HBV^−^ DLBCL (*n* = 70)	*p*
Sex						
Male	37 (52.9%)	226 (48.9%)	0.539	37 (52.9%)	33 (47.1%)	0.499
Female	33 (47.1%)	236 (51.1%)		33 (47.1%)	37 (52.9%)	
Age (years)						
<60	34 (48.6%)	224 (48.5%)	0.989	34 (48.6%)	32 (45.7%)	0.735
≥60	36 (51.4%)	238 (51.5%)		36 (51.4%)	38 (54.3%)	
B symptom						
Yes	21 (30.0%)	137 (29.7%)	0.953	21 (30.0%)	29 (41.4%)	0.158
No	49 (70.0%)	325 (70.3%)		49 (70.0%)	41 (58.6%)	
ECOG						
0–1	52 (74.3%)	320 (69.3%)	0.393	52 (74.3%)	40 (57.1%)	0.033^∗^
2–4	18 (25.7%)	142 (30.7%)		18 (25.7%)	30 (42.9%)	
Ann Arbor stage						
I–II	21 (30.0%)	195 (42.2%)	0.053	21 (30.0%)	25 (35.7%)	0.472
III–IV	49 (70.0%)	267 (57.8%)		49 (70.0%)	45 (64.3%)	
IPI score						
0–1	23 (32.9%)	173 (37.4%)	0.458	23 (32.9%)	19 (27.1%)	0.461
2–5	47 (67.1%)	289 (62.6%)		47 (67.1%)	51 (72.9%)	
AST						
≤ULN	59 (84.3%)	411 (89.0%)	0.256	59 (84.3%)	58 (82.9%)	0.820
>ULN	11 (15.7%)	51 (11.0%)		11 (15.7%)	12 (17.1%)	
ALT						
≤ULN	61 (87.1%)	433 (93.7%)	0.046^∗^	61 (87.1%)	59 (84.3%)	0.629
>ULN	9 (12.9%)	29 (6.3%)		9 (12.9%)	11 (15.7%)	
LDH						
≤ULN	36 (51.4%)	259 (56.1%)	0.467	36 (51.4%)	30 (42.9%)	0.310
>ULN	34 (48.6%)	203 (43.9%)		34 (48.6%)	40 (57.1%)	
β2-MG						
≤ULN	10 (14.3%)	90 (19.5%)	0.300	10 (14.3%)	12 (17.1%)	0.642
>ULN	60 (85.7%)	372 (80.5%)		60 (85.7%)	58 (82.9%)	
ESR						
≤20	32 (45.7%)	166 (35.9%)	0.115	32 (45.7%)	21 (30.0%)	0.055
>20	38 (54.3%)	296 (64.1%)		38 (54.3%)	49 (70.0%)	
Liver involvement						
Yes	6 (8.6%)	27 (5.8%)	0.378	6 (8.6%)	8 (11.4%)	0.573
No	64 (91.4%)	435 (94.2%)		64 (91.4%)	62 (88.6%)	
Spleen involvement						
Yes	19 (27.1%)	55 (11.9%)	<0.001^∗^	19 (27.1%)	16 (22.9%)	0.558
No	51 (72.9%)	407 (88.1%)		51 (72.9%)	54 (77.1%)	
Bone marrow involvement						
Yes	17 (24.3%)	51 (11.0%)	0.002^∗^	17 (24.3%)	16 (22.9%)	0.842
No	53 (75.7%)	411 (89.0%)		53 (75.7%)	54 (77.1%)	
Extranodal sites						
≤2	49 (70.0%)	376 (81.4%)	0.027^∗^	49 (70.0%)	53 (75.7%)	0.447
>2	21 (30.0%)	86 (18.6%)		21 (30.0%)	17 (24.3%)	
Bulky mass						
Yes	8 (11.4%)	43 (9.3%)	0.574	8 (11.4%)	9 (12.9%)	0.796
No	62 (88.6%)	419 (90.7%)		62 (88.6%)	61 (87.1%)	
Cell of origin						
GCB	18 (25.7%)	125 (27.1%)	0.120	18 (25.7%)	21 (30.0%)	0.094
ABC	52 (74.3%)	312 (67.5%)		52 (74.3%)	45 (64.3%)	
Uncategorized	0 (0.0%)	25 (5.4%)		0 (0.0%)	4 (5.7%)	
Ki-67						
<80%	41 (58.6%)	230 (49.8%)	0.171	41 (58.6%)	44 (62.9%)	0.604
≥80%	29 (41.4%)	232 (50.2%)		29 (41.4%)	26 (37.1%)	
Bcl2						
<70%	30 (42.9%)	252 (54.5%)	0.068	30 (42.9%)	42 (60.0%)	0.042^∗^
≥70%	40 (57.1%)	210 (45.5%)		40 (57.1%)	28 (40.0%)	
C-MYC						
<40%	42 (60.0%)	249 (53.9%)	0.339	42 (60.0%)	38 (54.3%)	0.495
≥40%	28 (40.0%)	213 (46.1%)		28 (40.0%)	32 (45.7%)	
DE						
Yes	18 (25.7%)	103 (22.3%)	0.541	18 (25.7%)	13 (18.6%)	0.416
No	52 (74.3%)	359 (77.7%)		52 (74.3%)	57 (81.4%)	
Assessment of efficacy						
PR	23 (32.9%)	106 (22.9%)	0.071	23 (32.9%)	18 (25.7%)	0.353
CR	47 (67.1%)	356 (77.1%)		47 (67.1%)	52 (74.3%)	

ECOG, Eastern Cooperative Oncology Group; IPI, International Prognostic Index; AST, aspartate transaminase; ALT, alanine aminotransferase; LDH, lactate dehydrogenase; ESR, erythrocyte sedimentation rate; DE: double-expressor; β2-MG, beta-2 microglobulin; and ULN, upper level of normal. ∗Significant *p* value (<0.05).

After PSM, the number of patients with ECOG scores of 2–4 was fewer in the HBV^+^ DLBCL group than in the HBV^−^ DLBCL group (*p* = 0.033), but the number of patients with Bcl-2 ≥70% was greater in the HBV^+^ DLBCL group than in the HBV^−^ DLBCL group (*p* = 0.042). There were no significant differences in the other variables (Table 1).

Seventy patients with HBV^+^ DLBCL were included in this study; among them, 30 agreed to chidamide maintenance treatment (chidamide group), and 40 declined (control group). The baseline characteristics of the patients are shown in Table 2. The chidamide group included a greater number of patients younger than 60 years (*p =* 0.032) and fewer patients with bone marrow involvement (*p =* 0.016) and the involvement of more than two extranodal sites (*p =* 0.008) than the control group. No significant differences were observed in terms of sex, B symptoms, ECOG score, Ann Arbor stage, IPI score, liver function, LDH, β2-MG, ESR, HBV load, other involved sites, cell origin, or pathological features.

**Table 2:** Clinical characteristics of patients with HBV^+^ DLBCL receiving chidamide maintenance therapy and the control group

Variables	HBV^+^ DLBCL (*n* = 70)	Chidamide (*n* = 30)	Control (*n* = 40)	*χ2*	*p*
Sex					
Male	37 (52.9%)	17 (56.7%)	20 (50.0%)	0.306	0.580
Female	33 (47.1%)	13 (43.3%)	20 (50.0%)		
Age (years)					
<60	34 (48.6%)	19 (63.3%)	15 (37.5%)	4.580	0.032^∗^
≥60	36 (51.4%)	11 (36.7%)	25 (62.5%)		
B symptom					
Yes	21 (30.0%)	11 (36.7%)	10 (25.0%)	1.111	0.292
No	49 (70.0%)	19 (63.3%)	30 (75.0%)		
ECOG					
0–1	52 (74.3%)	20 (66.7%)	32 (80.0%)	1.595	0.207
2–4	18 (25.7%)	10 (33.3%)	8 (20.0%)		
Ann Arbor stage					
I–II	21 (30.0%)	6 (20.0%)	15 (37.5%)	2.500	0.114
III–IV	49 (70.0%)	24 (80.0%)	25 (62.5%)		
IPI score					
0–1	23 (32.9%)	9 (30.0%)	14 (35.0%)	0.194	0.659
2–5	47 (67.1%)	21 (70.0%)	26 (65.0%)		
AST					
≤ULN	59 (84.3%)	26 (86.7%)	33 (82.5%)	0.225	0.635
>ULN	11 (15.7%)	4 (13.3%)	7 (17.5%)		
ALT					
≤ULN	63 (87.1%)	28 (93.3%)	33 (82.5%)	1.796	0.180
>ULN	9 (12.9%)	2 (6.7%)	7 (17.5%)		
LDH					
≤ULN	36 (51.4%)	15 (50.0%)	21 (52.5%)	0.043	0.836
>ULN	34 (48.6%)	15 (50.0%)	19 (47.5%)		
β2-MG					
≤ULN	10 (14.3%)	5 (16.7%)	5 (12.5%)	0.243	0.622
>ULN	60 (85.7%)	25 (83.3%)	35 (87.5)		
ESR					
≤20	32 (45.7%)	16 (53.3%)	16 (40.0%)	1.228	0.268
>20	38 (54.3%)	14 (46.7%)	24 (60.0%)		
Liver involvement					
Yes	6 (8.6%)	2 (6.7%)	4 (10.0%)	0.243	0.622
No	64 (91.4%)	28 (93.3%)	36 (90.0%)		
Spleen involvement					
Yes	19 (27.1%)	24 (80.0%)	27 (67.5%)	1.354	0.244
No	51 (72.9%)	6 (20.0%)	13 (32.5%)		
Bone marrow involvement					
Yes	17 (24.3%)	3 (10.0%)	14 (35.0%)	5.827	0.016^∗^
No	53 (75.7%)	27 (90.0%)	26 (65.0%)		
Extranodal sites					
≤2	49 (70.0%)	26 (86.7%)	23 (57.5%)	6.944	0.008^∗^
>2	21 (30.0%)	4 (13.3%)	17 (42.5%)		
Bulky disease					
Yes	8 (11.4%)	5 (16.7%)	3 (7.5%)	1.423	0.233
No	62 (88.6%)	25 (83.3%)	37 (92.5%)		
HBV DNA viral load					
High	25 (35.7%)	11 (36.7%)	14 (35.0%)	0.021	0.885
Low	45 (64.3%)	19 (63.3%)	26 (65.0%)		
Cellular origin					
GCB	18 (25.7%)	6 (20.0%)	12 (30.0%)	0.897	0.343
ABC	52 (74.3%)	24 (80.0%)	28 (70.0%)		
Ki-67					
<80	41 (58.6%)	18 (60.0%)	23 (57.5%)	0.044	0.834
≥80	29 (41.4%)	12 (40.0%)	17 (42.5%)		
BCL2^+^	40 (57.1%)	19 (63.3%)	21 (52.5%)	0.822	0.365
c-MYC^+^	28 (40.0%)	12 (40.0%)	16 (40.0%)	0.000	1.000
Assessment of efficacy					
PR	23 (32.9%)	9 (30.0%)	14 (35.0%)	0.194	0.659
CR	47 (67.1%)	21 (70.0%)	26 (65.0%)		

ECOG, Eastern Cooperative Oncology Group; IPI, International Prognostic Index; AST, aspartate transaminase; ALT, alanine aminotransferase; LDH, lactate dehydrogenase; ESR, erythrocyte sedimentation rate; β2-MG, beta-2 microglobulin; and ULN, upper level of normal. ∗Significant *p* value (<0.05).

### Therapeutic response and long-term outcomes

**Figure 2:**
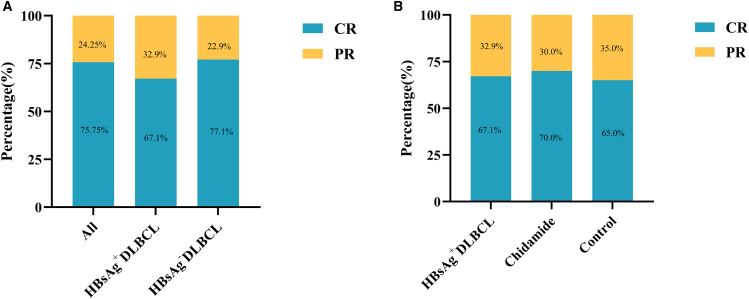
Therapeutic response between groups (A) Treatment response to first-line therapy in patients with HBV^+^ versus HBV^−^ DLBCL. (B) Treatment response in patients with HBV^+^ DLBCL receiving chidamide maintenance versus the control group following first-line therapy.

This study enrolled patients with DLBCL who achieved PR/CR after first-line therapy, and the treatment responses are summarized in Figure 2A. HBV^+^ DLBCL patient responses to first-line therapy in the chidamide and control groups are shown in Figure 2B. Overall, no significant difference in treatment response was observed between patients with HBV^+^ and HBV^−^ DLBCL. Similarly, among patients with HBV^+^ DLBCL, no significant difference was observed between the chidamide and control groups in terms of response evaluation after first-line treatment.

**Figure 3:**
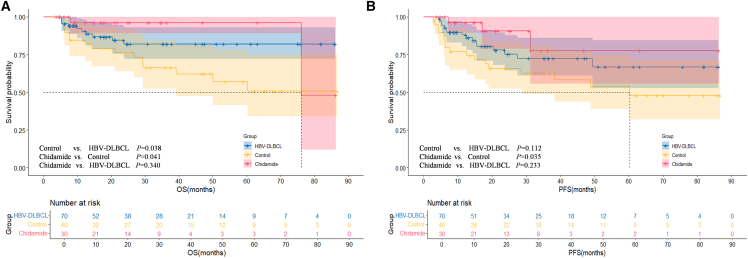
Long-term outcomes in patients with HBV^+^ and HBV^−^ DLBCL (A) OS outcomes of the HBV^−^ DLBCL, control, and chidamide groups. (B) PFS outcomes of the HBV^−^ DLBCL, control, and chidamide groups.

The deadline for follow-up was July 31, 2024. By the end of the follow-up period, 25.71% (36/140) of the entire cohort had experienced disease recurrence, disease progression, or death. The median follow-up time was 30.7 months. The 2-year OS rates of the HBV^−^ DLBCL and control groups were 86.8% and 66.4%, respectively, and the 3-year OS rates were 84.5% and 50.7%, respectively (*p =* 0.038). The 2- and 3-year PFS rates were 86.2% and 80.4%, respectively, in the HBV^−^DLBCL group and 62.5% and 47.9%, respectively, in the control group. Although no significant difference was observed between the two groups (*p =* 0.112), the HBV^−^DLBCL group showed a trend toward better PFS outcomes. In patients with HBV^+^ DLBCL, chidamide maintenance therapy significantly improved OS and PFS rates compared with those observed in the control group (*p* = 0.041 and *p* = 0.035, respectively), with 2-year OS rates of 96.2% versus 66.4% and 2-year PFS rates of 77.7% versus 62.5%. In addition, although no significant difference was observed in the OS and PFS rates between the chidamide and HBV^−^DLBCL groups (*p =* 0.340 and *p =* 0.233, respectively), a trend toward better outcomes was observed in the chidamide group (Figures 3A and 3B).

**Figure 4:**
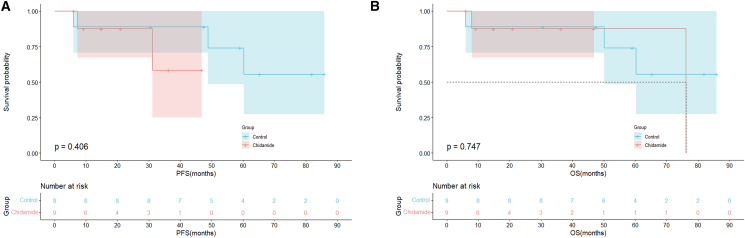
Long-term outcomes of chidamide maintenance therapy in patients with HBV^+^ DLBCL with double expression (A) PFS outcomes of the chidamide versus control groups. (B) OS outcomes of the chidamide versus control groups.

**Figure 5:**
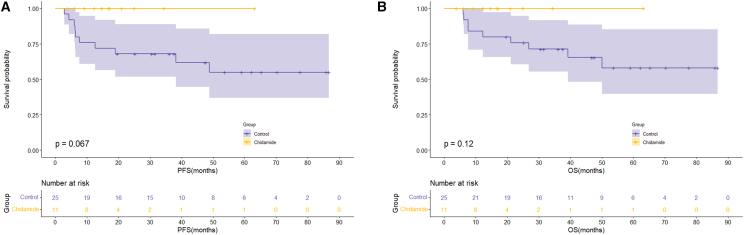
Long-term efficacy of chidamide maintenance therapy in elderly (≥60 years) patients with HBV^+^ DLBCL (A) PFS outcomes of the chidamide versus control groups. (B) OS outcomes of the chidamide versus control groups.

**Figure 6:**
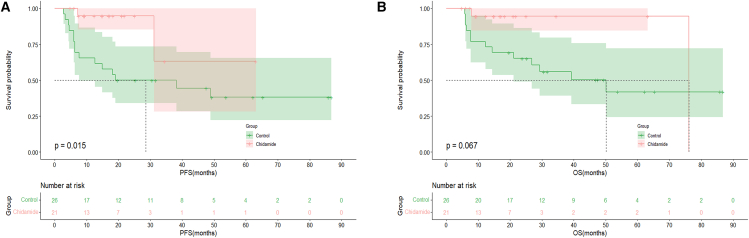
Long-term efficacy of chidamide maintenance therapy in patients with HBV^+^ DLBCL with an IPI score of 2–5 (A) PFS outcomes of the chidamide versus control groups. (B) OS outcomes of the chidamide versus control groups.

In the subgroup analysis of patients with HBV^+^ DLBCL, no significant differences in PFS or OS outcomes were observed between patients with MYC/BCL2 double-expression status in the chidamide and control groups (Figures 4A and 4B; OS: *p =* 0.747; PFS: *p =* 0.406). Similarly, in elderly patients (aged ≥60 years), the PFS and OS outcomes were comparable between the two groups (Figures 5A and 5B; PFS: *p* = 0.067; OS: *p =* 0.120). Among patients with an IPI score of 2–5, PFS rates were superior in the chidamide versus control group, and the difference was significant (Figure 6A; PFS: *p* = 0.015). The median OS times were 76.1 months in the chidamide group and 50.1 months in the control group; however, the difference was not significant (Figure 6B; OS: *p* = 0.067).

### Univariate and multivariate analyses of overall survival and progression-free survival outcomes

**Figure 7:**
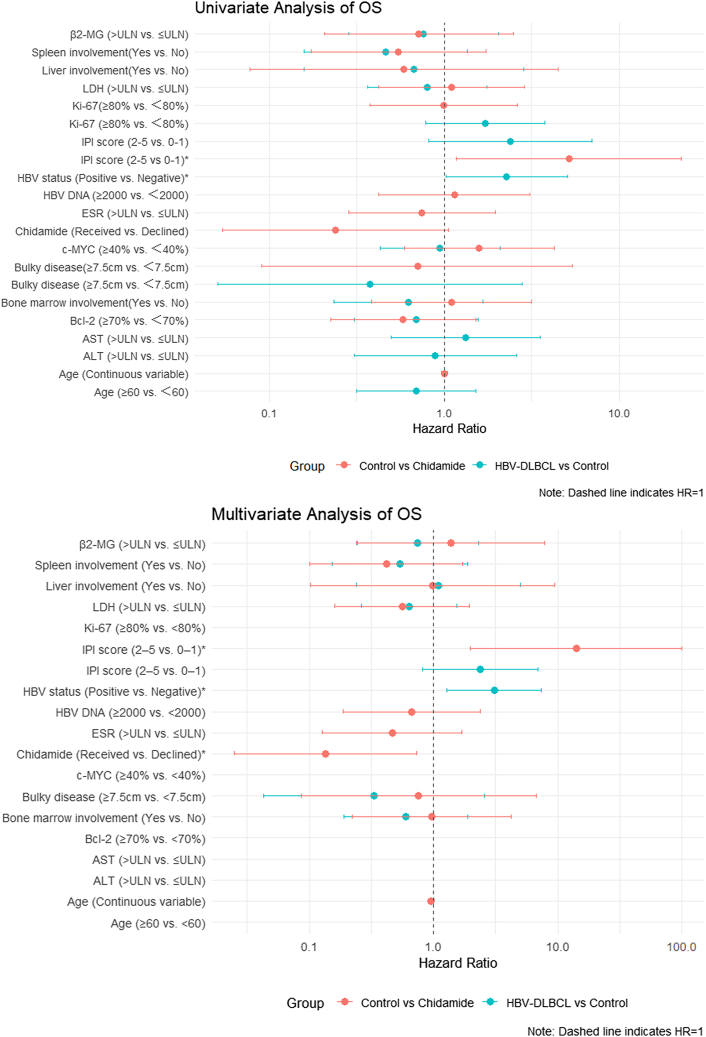
Univariate and multivariate regression analyses of OS outcomes IPI, International Prognostic Index; AST, aspartate transaminase; ALT, alanine aminotransferase; LDH, lactate dehydrogenase; ESR, erythrocyte sedimentation rate; β2-MG, beta-2 microglobulin; and ULN, upper level of normal. ∗Significant *p* value (<0.05).

**Figure 8:**
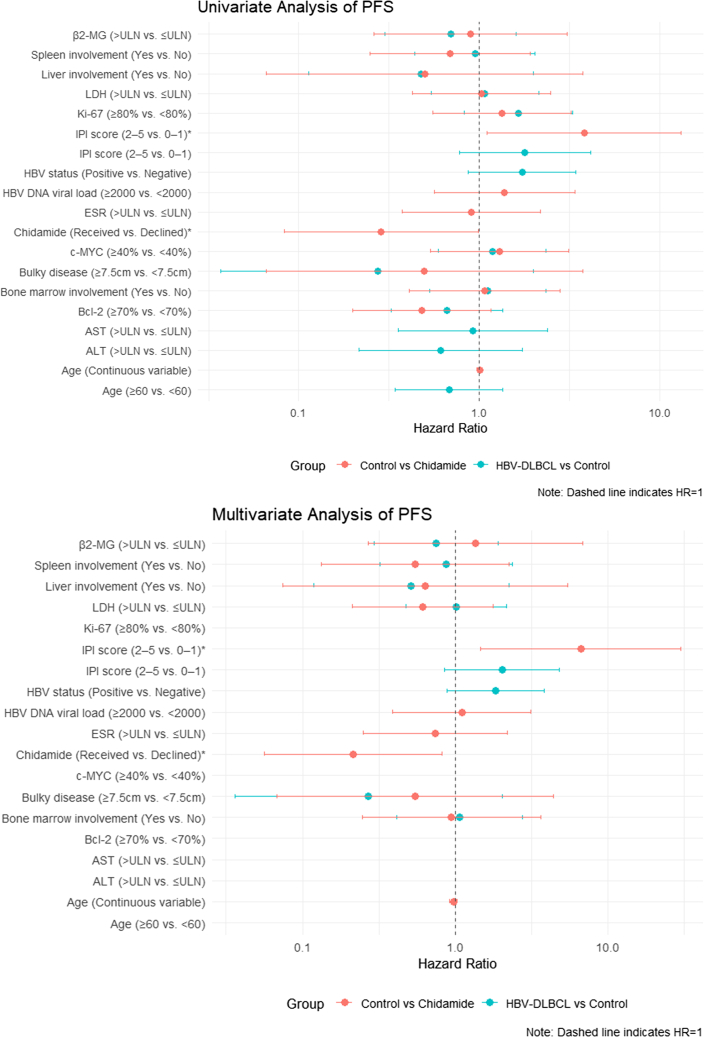
Univariate and multivariate regression analyses of PFS outcomes IPI, International Prognostic Index; AST, aspartate transaminase; ALT, alanine aminotransferase; LDH, lactate dehydrogenase; ESR, erythrocyte sedimentation rate; β2-MG, beta-2 microglobulin; and ULN, upper level of normal. ∗Significant *p* value (<0.05).

The following covariates were included in the Cox regression analysis: HBV status; age; IPI score; LDH, β2-MG, and ESR levels; Ki-67, Bcl-2, and c-MYC expression; different sites of involvement and bulky disease; and whether chidamide maintenance therapy was administered (Figures 7 and 8). Given the younger age distribution in the chidamide group, we adjusted for age as a continuous variable in the Cox regression analysis to mitigate potential age-related bias and ensure the robustness of the study conclusions. In the multivariate regression analysis, poor long-term outcomes (OS) were associated with HBV infection (OS: HR [95% CI] = 3.078 [1.284, 7.377], *p* = 0.012) in the HBV^−^ DLBCL versus control group comparison.

Regarding the control and chidamide groups, better long-term outcomes (PFS and OS) were associated with receiving chidamide maintenance therapy (OS: HR [95% CI] = 0.135[0.025, 0.738], *p* = 0.021; PFS: HR [95% CI] = 0.215 [0.056, 0.823], *p* = 0.025). Furthermore, an IPI score of ≥2 (OS: HR [95% CI] = 14.057 [1.975, 100.034], *p* = 0.008; PFS: HR [95% CI] = 6.657 [1.473, 30.087], *p* = 0.014) was an independent risk factor for OS and PFS.

### Hepatitis B virus-related events

Among the patients with HBV^+^ DLBCL, 25 (35.7%) had a baseline HBV viral load of ≥2000 IU/mL, of whom 11 (36.4%) and 19 (63.3%) had a high HBV viral load in the chidamide and control groups, respectively (Table 2). No HBV reactivation-related events, fatal HBV-related liver failure, or liver cancer occurred during the follow-up period.

### Safety

Chidamide maintenance therapy was generally well tolerated. None of the patients underwent treatment reduction or discontinuation due to adverse reactions. The most common hematologic toxicities were neutropenia (all grades, 63.33%), thrombocytopenia (all grades, 53.33%), and anemia (all grades, 43.33%). The most common nonhematologic toxicities were anorexia (all grades, 36.67%) and fatigue (all grades, 23.33%). The main serious AEs (grade ≥3) were hematological, especially neutropenia, which improved after timely treatment. AEs occurred mainly in the first 6 months and gradually decreased in frequency thereafter (Table 3).

**Table 3:** Adverse events (AEs) of chidamide (*n* = 30)

Toxicity type		Any grade, n (%)	Grade ≥3, n (%)
Hematologic AEs	Neutropenia	29 (63.33)	4 (13.33)
	Anemia	13 (43.33)	1 (3.33)
	Thrombocytopenia	16 (53.33)	3 (10.00)
	Elevated liver enzymes	0 (0.00)	0 (0.00)
	Renal dysfunction	0 (0.00)	0 (0.00)
	Electrolyte disturbance	3 (10.00)	0 (0.00)
Nonhematologic AEs	Nausea/vomiting	4 (13.33)	0 (0.00)
	Anorexia	11 (36.67)	0 (0.00)
	Fever	0 (0.00)	0 (0.00)
	Allergy	1 (3.33)	0 (0.00)
	Fatigue	7 (23.33)	0 (0.00)

## Discussion

Despite achieving CR or PR following standard first-line treatment, patients with HBV^+^ DLBCL continue to experience suboptimal long-term outcomes. Previous studies have demonstrated that HBV infection is associated with increased therapeutic resistance, higher relapse rates, and inferior survival in patients with DLBCL, highlighting the need for more effective post-remission strategies in this high-risk subgroup.

In this context, our study was designed as a single-center, single-arm, open-label clinical trial to investigate the efficacy and safety of chidamide as a maintenance therapy for patients with HBV^+^ DLBCL who achieved CR or PR after induction therapy. Although our analysis included comparisons with patients who did not receive chidamide therapy, the study remained methodologically single-arm in nature. All patients in the primary cohort received chidamide, whereas the control group was retrospectively included to provide contextual reference, rather than serving as a randomized or prospectively assigned control arm.

Although numerous studies have explored the mechanisms underlying HBV-associated lymphoma, our understanding remains limited, which is partly due to the complexity of the HBV life cycle itself and the difficulty of modeling HBV-associated lymphoma. Direct mechanisms, such as intracellular HBV DNA-mediated carcinogenesis, and indirect mechanisms, such as the extracellular HBV stimulation of B lymphocytes, are involved^26^; thus, additional basic research is needed for mechanistic clarification. Several epidemiological and clinical studies have suggested that DLBCL coinfected with HBV may constitute a distinct subgroup with different clinical features.^11^^,^^27^^,^^28^ In this study, more HBV^+^ DLBCL than patients with HBV^−^ DLBCL presented a greater frequency of abnormal liver function and splenic involvement, consistent with the results of a previous study.^29^ Our finding is that a greater frequency of bone marrow involvement was observed in patients with HBV^+^ DLBCL than in patients with HBV^−^ DLBCL, suggesting that HBV^+^ DLBCL may have a greater capacity for migration and invasion.

The current mainstream opinion is that patients with HBV-infected DLBCL have significantly poor OS and PFS outcomes^15^; in our study, it was confirmed that the OS rate of patients with HBV^+^ DLBCL was worse than that of those with HBV^−^ DLBCL. Although no statistical difference was observed in the PFS rate, the PFS outcome of patients with HBV^−^ DLBCL showed a trend toward improvement, which may have been limited by the small sample size. Researchers believe that HBV infection contributes to genomic instability, immune evasion, resistance to first-line chemotherapy, and, critically, epigenetic dysregulation, which collectively underlie the poor prognosis observed in DLBCL patients with HBV infection.^19^^,^^30^ Among these mechanisms, epigenetic alterations have emerged as a pivotal link between chronic viral infection and lymphomagenesis. HBV has been shown to interfere with host epigenetic machinery, particularly by modulating histone modifications and DNA methylation, thereby promoting oncogenic transcriptional programs.^31^^,^^32^ In parallel, DLBCL is characterized by frequent epigenetic aberrations, with a substantial subset of patients (25%) harboring loss-of-function mutations in genes encoding key histone acetyltransferases, such as *CREBBP* and *EP300*.^33^^,^^34^ These insights underscore the rationale for incorporating epigenetic therapies into DLBCL regimens, particularly in the context of HBV coinfection.

Chidamide, a selective HDACi, was first approved by the China National Medical Products Administration in 2014 for the treatment of relapsed or refractory PTCL. In 2024, it was also approved for previously untreated double-expressor DLBCL (MYC^+^/BCL2^+^).^35^ With the growing recognition of HBV’s influence on epigenetic regulation, especially its modulation of histone deacetylation, the potential for using chidamide in the treatment of HBV-associated DLBCL has become increasingly apparent. Notably, existing research has confirmed the interaction between HBV and HDACs, including the effect of the HBx protein on cell cycle regulation through epigenetic control^36^ and the change in HBV replication products related to histone deacetylation.^37^ While much of this research has concentrated on liver disease, its implications for treating HBV-related lymphoma are substantial. These findings provide robust support for the use of chidamide in maintenance therapy for HBV-related DLBCL, particularly given that first-line treatments fail to meet treatment expectations for this condition. Our study confirmed that the administration of chidamide maintenance therapy to patients with HBV^+^ DLBCL who achieved PR/CR on first-line therapy could overcome the adverse outcomes of HBV infection, resulting in similar PFS and OS rates for these patients as those observed in patients with HBV^−^ DLBCL. In the subgroup analysis of patients with HBV-positive DLBCL, chidamide maintenance therapy was associated with improved PFS in those with intermediate-to-high IPI scores (2–5). Although no significant difference in OS was observed, the chidamide group showed a favorable trend. These findings suggest that chidamide maintenance therapy may have particular clinical relevance for patients with higher baseline risk. However, given the limited sample size in this subgroup, these results should be interpreted with caution and warrant further validation in prospective studies. Notably, despite the younger age distribution observed in the chidamide maintenance therapy group, we rigorously addressed potential age-related confounding by incorporating age as a continuous variable in multivariable Cox proportional hazards models. Crucially, chidamide maintenance therapy retained its significant association with survival benefits after comprehensive adjustment. These robust findings underscore that the therapeutic efficacy of chidamide is independent of demographic variations, highlighting its clinical promise for optimized maintenance strategies. Moreover, chidamide maintenance therapy has demonstrated excellent safety and tolerability, providing insights for further improving the prognosis of patients with HBV^+^ DLBCL and offering an additional approach for the treatment of virus-associated lymphoma.

### Limitations and future directions

This study also has several limitations. As a single-center study, our HBV^+^ DLBCL patient population was relatively small and underrepresented, reflecting a slight limitation in sample complexity and diversity. Moreover, this single-arm clinical study has the potential for bias, as the small sample size resulted in a minor inadequacy in our assessment of rare serious AEs, which increased the uncertainty in the interpretation of data on drug safety and efficacy. As an open clinical trial, there may be a potential imbalance. In the future, we look forward to more centers joining the study to provide additional data support for chidamide maintenance therapy. We will also explore the mechanism of chidamide maintenance therapy for HBV^+^ DLBCL in future basic experiments to provide more mechanistic insights into the advantages of chidamide maintenance therapy.

## Resource availability

### Lead contact

Further information and requests for resources and reagents should be directed to and will be fulfilled by the lead contact, Dr. Ou Bai (baiou@jlu.edu.cn).

### Materials availability

This study did not generate new materials.

### Data and code availability

• **Data**: The datasets generated in this study have been deposited in **Mendeley Data** and are publicly available at: https://doi.org/10.17632/vpgzftby6w.1. The dataset is titled *“Chidamide: A new exploration of maintenance therapy for DLBCL patients with HBV infection”* (Ying Zhang).
• **Code**: No new code was generated in this study. Any scripts used for data analysis are available from the lead contact upon reasonable request.
• **Other items**: This article has no additional information or items beyond those stated above.

## Acknowledgments

This work was supported by the 10.13039/501100009991Department of Finance of Jilin Province (JLSWSRCZX2023-8) and the 10.13039/501100002855Ministry of Science and Technology of the People's Republic of China–Foreign Experts Project (G2023129019L).

## Author contributions

Y.Z.: writing—original draft and review and editing and validation. H.T.: writing – review and editing, and visualization. W.G.: investigation and writing – review and editing. Y.Z.: data collection and writing – review and editing. Z.Z.: investigation and writing – review and editing. Z.L.: analysis and writing – review and editing. B.W.: conceptualization and writing –review and editing. O.B.: supervision and writing – review and editing.

## Declaration of interests

The authors declare that the research was conducted in the absence of any commercial or financial relationships that could be construed as potential conflicts of interest.

## STAR★Methods

### Key resources table

**Table:** 

REAGENT or RESOURCE	SOURCE	IDENTIFIER
**Deposited data**		

Raw de-identified clinical data	Mendeley Data	https://doi.org/10.17632/vpgzftby6w.1

**Software and algorithms**		

R 4.3.1	R package	https://www.r-project.org/.
GraphPad Prism 8.0 software	Chi-square test	https://www.graphpad.com/features.

### Experimental model and study participant details

#### Human subjects

All participants were human subjects diagnosed with DLBCL. As the study was conducted in northern China, all enrolled individuals were of East Asian ancestry. No cell lines, animals, plants, or microbial strains were used in this study.

#### Study population and eligibility

This study was a single-arm, open-label, phase II clinical trial conducted at The First Hospital of Jilin University to evaluate the efficacy and safety of chidamide maintenance therapy in HBV-positive DLBCL patients. Eligible participants were newly diagnosed HBV-positive DLBCL patients treated at our center between June 2017 and June 2024. Both males and females were included, aged 18–85 years. Diagnoses were confirmed according to the 2016 WHO classification. All patients had completed 4–6 cycles of R-CHOP or CHOP-like regimens, and their treatment responses were assessed as CR or PR prior to enrollment for chidamide maintenance therapy.

Exclusion criteria included transformation from low-grade B-cell lymphoma, primary CNS DLBCL, co-infection with HCV/HIV/EBV/CMV, prior malignancy, or receipt of fewer than two cycles of chidamide maintenance therapy.

#### Sex and gender considerations

Both sexes were represented in the study. The study was not specifically powered to detect sex- or gender-related effects.

#### Sample size and allocation

A total of 30 HBV^+^ DLBCL patients received chidamide maintenance and were included as the treatment cohort.

Two comparison groups were defined:

(1) 40 contemporaneous HBV^+^ DLBCL patients who declined chidamide maintenance (real-world control).
(2) 70 HBV^-^ DLBCL patients matched 1:1 to HBV^+^ cases using propensity score matching (PSM).

### Method details

#### Sample size estimation

Sample size was calculated based on an anticipated objective response rate (ORR) of 70%, informed by prior studies in which chidamide combined with rituximab achieved ∼40% ORR in elderly patients with relapsed/refractory DLBCL. With a two-sided α = 0.05 and 80% power, a minimum of 24 patients was required. Considering a 10% loss to follow-up, the planned enrollment was 27 participants.

#### Treatment procedures

Patients receiving chidamide maintenance therapy were administered 20 mg orally twice weekly for up to 24 months, or until disease progression, unacceptable toxicity, withdrawal of consent, or death. Treatment interruptions or dose reductions were performed according to established clinical management guidelines.

Patients were evaluated every 3 months with complete blood counts, serum chemistry, liver and renal function tests, HBV-DNA, and radiologic assessments. Adverse events (AEs) were documented and graded according to CTCAE v6.0.

#### HBV management

HBV-DNA quantification and full HBV serology were obtained at baseline. Antiviral prophylaxis (lamivudine or entecavir) was administered with high-risk chemotherapy regimens and continued for at least six months after therapy completion.

#### HBV^-^ DLBCL matched cohort

HBV-negative DLBCL patients were matched 1:1 to HBV^+^ DLBCL cases using propensity score matching (PSM) to assess whether chidamide maintenance could mitigate the adverse prognostic effects associated with HBV infection.

#### Propensity score matching (PSM)

Propensity scores were estimated using a logistic regression model incorporating the following baseline covariates: gender, age, cell-of-origin subtype, IPI score, ALT level, liver involvement, spleen involvement, bone marrow involvement, and Extranodal sites>2.

Matching was performed using nearest-neighbor matching without replacement with a 1:1 ratio and a caliper of 0.2 SD of the logit of the propensity score. Covariate balance was assessed via standardized mean differences (SMD) with SMD <0.1 defined as acceptable.

Detailed matching outputs are provided in Supplementary File 1.

#### Response assessment and clinical definitions

Follow-up continued until July 31, 2024 or death.

Tumor response was evaluated according to the 2007 Revised Response Criteria for Malignant Lymphoma. OS: time from diagnosis to death from any cause or last follow-up. PFS: time from diagnosis to relapse, progression, death, or last follow-up. HBV-DNA levels: categorized as low: <2,000 IU/mL; high:≥2,000 IU/mL. HBV reactivation was defined as a ≥2-log_10_ increase from baseline or the emergence of HBsAg in previously HBsAg–/HBcAb+ individuals. Liver dysfunction: AST or ALT >1.25 × ULN. Double-expressor lymphoma (DEL): MYC ≥40% and BCL2 ≥50% by immunohistochemistry.

### Quantification and statistical analysis

Categorical variables were analyzed using chi-square or Fisher’s exact tests. Kaplan–Meier curves were used to estimate OS and PFS, with differences assessed by log-rank tests. Univariate Cox regression was used to identify candidate prognostic variables, which were then included in multivariate Cox proportional hazards models. Hazard ratios (HRs) and 95% confidence intervals (CIs) were reported.

A two-sided p-value <0.05 was considered statistically significant.

### Additional resources

This clinical study is registered at ClinicalTrials.gov under identifier NCT04661943(https://clinicaltrials.gov/ct2/show/NCT04661943).

## References

[1] Lipsick J. (2021). A History of Cancer Research: Tumor Viruses. Cold Spring Harb. Perspect. Biol..

[2] Chen C.J., You S.L., Hsu W.L., Yang H.I., Lee M.H., Chen H.C., Chen Y.Y., Liu J., Hu H.H., Lin Y.J. (2021). Epidemiology of Virus Infection and Human Cancer. Recent Results Cancer Res..

[3] Chen J., Wang J., Yang J., Zhang W., Song X., Chen L. (2013). Concurrent infection of hepatitis B virus negatively affects the clinical outcome and prognosis of patients with non-Hodgkin's lymphoma after chemotherapy. PLoS One.

[4] Kim J.H., Bang Y.J., Park B.J., Yoo T., Kim C.W., Kim T.Y., Heo D.S., Lee H.S., Kim N.K. (2002). Hepatitis B virus infection and B-cell non-Hodgkin's lymphoma in a hepatitis B endemic area: a case-control study. Jpn. J. Cancer Res..

[5] Takai S., Tsurumi H., Ando K., Kasahara S., Sawada M., Yamada T., Hara T., Fukuno K., Takahashi T., Oyama M. (2005). Prevalence of hepatitis B and C virus infection in haematological malignancies and liver injury following chemotherapy. Eur. J. Haematol..

[6] Zhou X., Pan H., Yang P., Ye P., Cao H., Zhou H. (2019). Both chronic HBV infection and naturally acquired HBV immunity confer increased risks of B-cell non-Hodgkin lymphoma. BMC Cancer.

[7] Huang X., Young K.H., Guo W., Wang Y., Wang X., Xi Y., Wang L., Bai O. (2020). Identification of hepatitis B virus aetiologic antigens, HBx and Pre-S2, in diffuse large B-cell lymphoma. J. Viral Hepat..

[8] Ren W., Ye X., Su H., Li W., Liu D., Pirmoradian M., Wang X., Zhang B., Zhang Q., Chen L. (2018). Genetic landscape of hepatitis B virus-associated diffuse large B-cell lymphoma. Blood.

[9] Dalia S., Chavez J., Castillo J.J., Sokol L. (2013). Hepatitis B infection increases the risk of non-Hodgkin lymphoma: a meta-analysis of observational studies. Leuk. Res..

[10] Zhang Y., Guo W., Zhan Z., Bai O. (2024). Carcinogenic mechanisms of virus-associated lymphoma. Front. Immunol..

[11] Zhang M.Y., Gao F., Zhao Y.W., Ni B.W., Huang H.H., Hou J. (2022). Inferior survival and frequent hepatic dysfunction in non-Hodgkin's lymphoma patients with HBV infection: a systematic review and meta-analysis. Hematology.

[12] Deng L., Song Y., Young K.H., Hu S., Ding N., Song W., Li X., Shi Y., Huang H., Liu W. (2015). Hepatitis B virus-associated diffuse large B-cell lymphoma: unique clinical features, poor outcome, and hepatitis B surface antigen-driven origin. Oncotarget.

[13] Yan X., Zhou M., Lou Z., Mu Q., Sheng L., Zhang P., Wang Y., Ouyang G. (2018). Diffuse large B-cell lymphoma with concurrent hepatitis B virus infection in the MabThera era: Unique clinical features and worse outcomes. J. Cancer Res. Ther..

[14] Yang W., Zhao X., Ma H., Xu C. (2024). Real-world clinical features and survival outcomes in diffuse large B-cell lymphoma patients with hepatitis B virus infection. Infect. Agent. Cancer.

[15] Rong X., Wang H., Ma J., Pan S., Wang H., Jing S., Su Y., Wang L., Zhao C. (2019). Chronic hepatitis B virus infection is associated with a poorer prognosis in diffuse large B-cell lymphoma: a meta-analysis and systemic review. J. Cancer.

[16] Fu K., Weisenburger D.D., Choi W.W.L., Perry K.D., Smith L.M., Shi X., Hans C.P., Greiner T.C., Bierman P.J., Bociek R.G. (2008). Addition of rituximab to standard chemotherapy improves the survival of both the germinal center B-cell-like and non-germinal center B-cell-like subtypes of diffuse large B-cell lymphoma. J. Clin. Oncol..

[17] Wei Z., Zou S., Li F., Cheng Z., Li J., Wang J., Wang C., Chen F., Cao J., Cheng Y. (2014). HBsAg is an independent prognostic factor in diffuse large B cell lymphoma patients in rituximab era: result from a multicenter retrospective analysis in China. Med. Oncol..

[18] Zhao X., Guo X., Xing L., Yue W., Yin H., He M., Wang J., Yang J., Chen J. (2018). HBV infection potentiates resistance to S-phase arrest-inducing chemotherapeutics by inhibiting CHK2 pathway in diffuse large B-cell lymphoma. Cell Death Dis..

[19] Zhan Z., Yang W., Guo W., Wan X., Li J., Zhang Y., Wang B., Liang X., Bai O. (2024). HBx induces chemoresistance in diffuse large B cell lymphoma by inhibiting intrinsic apoptosis via the NF-kappaB/XIAP pathway. Mol. Ther. Nucleic Acids.

[20] Reddy N.M., Thieblemont C. (2017). Maintenance therapy following induction chemoimmunotherapy in patients with diffuse large B-cell lymphoma: current perspective. Ann. Oncol..

[21] You M., Gao Y., Fu J., Xie R., Zhu Z., Hong Z., Meng L., Du S., Liu J., Wang F.S. (2023). Epigenetic regulation of HBV-specific tumor-infiltrating T cells in HBV-related HCC. Hepatology.

[22] Rongrui L., Na H., Zongfang L., Fanpu J., Shiwen J. (2014). Epigenetic mechanism involved in the HBV/HCV-related hepatocellular carcinoma tumorigenesis. Curr. Pharm. Des..

[23] Dandri M. (2020). Epigenetic modulation in chronic hepatitis B virus infection. Semin. Immunopathol..

[24] Cao H.Y., Li L., Xue S.L., Dai H.P. (2023). Chidamide: Targeting epigenetic regulation in the treatment of hematological malignancy. Hematol. Oncol..

[25] Ridwansyah H., Wijaya I., Bashari M.H., Sundawa Kartamihardja A.H., Hernowo B.S. (2023). The role of chidamide in the treatment of B-cell non-Hodgkin lymphoma: An updated systematic review. Biomol. Biomed..

[26] Zhang W., Du F., Wang L., Bai T., Zhou X., Mei H. (2023). Hepatitis Virus-associated Non-hodgkin Lymphoma: Pathogenesis and Treatment Strategies. J. Clin. Transl. Hepatol..

[27] Zhang L., Yuan X.L., Jiang L., Yang J., Guo J.M., Shi J., Lei P.C., Zhang Y., Zhu Z.M. (2018). [Analysis of clinical characteristics and prognostic factors in patients with non-Hodgkin lymphoma and HBV infection]. Zhonghua Xue Ye Xue Za Zhi.

[28] Zhang Y.T., Feng D., Wang Y., Li D.P., Li Z.Y., Qiu T.T., Xu K.L. (2020). [Expression and Clinical Significance of EZH2 in Patients with Diffuse Large B Cell Lymphoma Accompanied by HBV Infection]. Zhongguo Shi Yan Xue Ye Xue Za Zhi.

[29] Chen D.G., Chen G., Wang C., Ke L.F., Wu H., He H.M., Yang Y., Chen Y.P. (2021). Clinicopathological and prognostic features of hepatitis B virus-associated diffuse large B-cell lymphoma: a single-center retrospective study in China. Infect. Agent. Cancer.

[30] Wan X., Young K.H., Bai O. (2023). HBV-associated DLBCL of poor prognosis: advance in pathogenesis, immunity and therapy. Front. Immunol..

[31] Jiang Y., Han Q., Zhao H., Zhang J. (2021). The Mechanisms of HBV-Induced Hepatocellular Carcinoma. J. Hepatocell. Carcinoma.

[32] Tan X., Xun L., Yin Q., Chen C., Zhang T., Shen T. (2025). Epigenetic Modifications in HBV-Related Hepatocellular Carcinoma. J. Viral Hepat..

[33] Morin R.D., Mendez-Lago M., Mungall A.J., Goya R., Mungall K.L., Corbett R.D., Johnson N.A., Severson T.M., Chiu R., Field M. (2011). Frequent mutation of histone-modifying genes in non-Hodgkin lymphoma. Nature.

[34] Pasqualucci L., Dominguez-Sola D., Chiarenza A., Fabbri G., Grunn A., Trifonov V., Kasper L.H., Lerach S., Tang H., Ma J. (2011). Inactivating mutations of acetyltransferase genes in B-cell lymphoma. Nature.

[35] Shi Y., Dong M., Hong X., Zhang W., Feng J., Zhu J., Yu L., Ke X., Huang H., Shen Z. (2015). Results from a multicenter, open-label, pivotal phase II study of chidamide in relapsed or refractory peripheral T-cell lymphoma. Ann. Oncol..

[36] Andrisani O.M. (2013). Deregulation of epigenetic mechanisms by the hepatitis B virus X protein in hepatocarcinogenesis. Viruses.

[37] Zhang D., Wang Y., Zhang H.Y., Jiao F.Z., Zhang W.B., Wang L.W., Zhang H., Gong Z.J. (2019). Histone deacetylases and acetylated histone H3 are involved in the process of hepatitis B virus DNA replication. Life Sci..

