# Supplementary material for: Chidamide: Exploration of maintenance therapy for patients with DLBCL with HBV infection
Source: iScience. 2025 Dec 1;29(1):114302. doi: 10.1016/j.isci.2025.114302 (PMC12800633; doi:10.1016/j.isci.2025.114302)
Supplement: Document S1. Figure S1 and Table S1 [file mmc1.pdf]

iScience, Volume *29*

Supplemental information

Chidamide: Exploration of maintenance therapy for patients with DLBCL with HBV infection

Ying Zhang, Haotian Wang, Wei Guo, Yangzhi Zhao, Zhumei Zhan, Zhe Li, Bowen Wang, and Ou Bai

Supplementary Table 1. Standardized Mean Differences (SMD) After Propensity Score Matching

	Type	Diff.Un	M.Threshold.Un
distance	Distance	0.065361	Balanced, <0.1
Gender	Binary	-0.05714	Balanced, <0.1
Age	Binary	-0.02857	Balanced, <0.1
COO	Binary	-0.1	Not Balanced, >0.1
IPI	Binary	-0.05714	Balanced, <0.1
ALT	Binary	-0.02857	Balanced, <0.1
Liver involvement	Binary	-0.02857	Balanced, <0.1
Spleen involvement	Binary	0.042857	Balanced, <0.1
Bone marrow involvement	Binary	0.014286	Balanced, <0.1
Extranodal sites > 2	Binary	0.057143	Balanced, <0.1

Although COO subtype showed an SMD of 0.1000, this borderline imbalance was considered acceptable for downstream analysis.

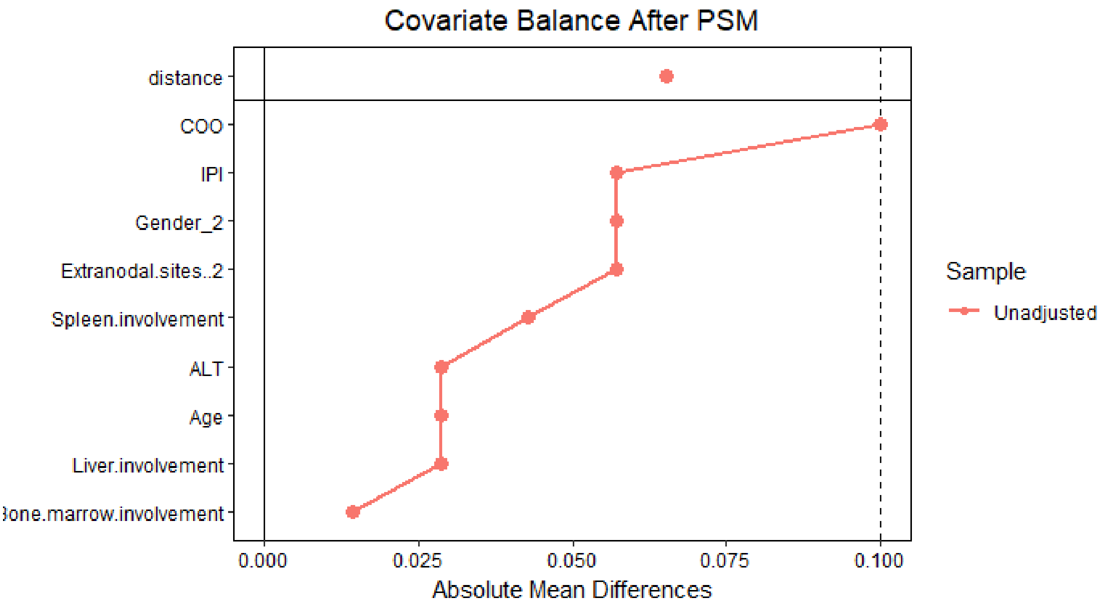

Supplementary Figure 1. Love plot demonstrating covariate balance after propensity score matching.
